# Study on emotion recognition bias in different regional groups

**DOI:** 10.1038/s41598-023-34932-z

**Published:** 2023-05-24

**Authors:** Martin Lukac, Gulnaz Zhambulova, Kamila Abdiyeva, Michael Lewis

**Affiliations:** Department of Computer Science, Nazarbayev University, Kabanbay Batyr 53, Astana, 010000 Kazakhstan

**Keywords:** Psychology, Human behaviour, Computational biology and bioinformatics

## Abstract

Human-machine communication can be substantially enhanced by the inclusion of high-quality real-time recognition of spontaneous human emotional expressions. However, successful recognition of such expressions can be negatively impacted by factors such as sudden variations of lighting, or intentional obfuscation. Reliable recognition can be more substantively impeded due to the observation that the presentation and meaning of emotional expressions can vary significantly based on the culture of the expressor and the environment within which the emotions are expressed. As an example, an emotion recognition model trained on a regionally-specific database collected from North America might fail to recognize standard emotional expressions from another region, such as East Asia. To address the problem of regional and cultural bias in emotion recognition from facial expressions, we propose a meta-model that fuses multiple emotional cues and features. The proposed approach integrates image features, action level units, micro-expressions and macro-expressions into a multi-cues emotion model (MCAM). Each of the facial attributes incorporated into the model represents a specific category: fine-grained content-independent features, facial muscle movements, short-term facial expressions and high-level facial expressions. The results of the proposed meta-classifier (MCAM) approach show that a) the successful classification of regional facial expressions is based on non-sympathetic features b) learning the emotional facial expressions of some regional groups can confound the successful recognition of emotional expressions of other regional groups unless it is done from scratch and c) the identification of certain facial cues and features of the data-sets that serve to preclude the design of the perfect unbiased classifier. As a result of these observations we posit that to learn certain regional emotional expressions, other regional expressions first have to be “forgotten”.

## Introduction

A significant component of human communication is non-verbal: facial expressions^1^, gestures, body posture, verbal emotional expressions^2,3^ and behavioral actions contribute up to 60% of the total communication^4,5^. Thus, an intelligent agent that can effectively interpret human non-verbal communication clues should also be able to better (faster, more accurately) interpret human verbal communication. Among the non-verbal communication components, facial expressions play a crucial role in the understanding of emotion^6^. Automated facial expression recognition (FER) is a non-invasive method of detecting emotions from images^7^. FER can be performed using a sequence of video frames for video-based recognition^8–10^. However, most approaches use still images extracted from video frames.

The versatility of human emotional expression is dependent on context, task and social environment. Interpreting emotional expressions without context is possible, but not sufficient to fully comprehend the extent of information conveyed^11^. Emotional understanding is very strongly affected by cultural and regional context^12^. For example, an emotion recognition model trained on a regionally-specific database, collected from North America, might fail to recognize standard emotional expressions from another region, such as East Asia. Regional dependency affects the recognition of facial emotional expressions in humans. For instance in^13^ the authors demonstrate that individuals within a regional group exhibits strong preferences when accurately recognizing facial emotional expressions. Several researchers collected data from regionally-specific areas, such as FER2013^14^ and JAFFE^15^. The FER2013 dataset is mostly composed of Caucasian and African-American individuals expressing emotions in an unposed manner. JAFFE is a database of Japanese female facial expressions with posed emotions. As expected, due to the differing regional contexts, systems that are trained on the FER2013^14^ dataset performed poorly when evaluated on the JAFFE^15^ dataset, and vice-versa.

While one could argue that such a difference of performance is due to the posed and unposed emotional expressions, several works have demonstrated that the emotional expressions might not be as universal as expected^16^. Additionally, a FER model, trained on a specific dataset, exploits a variety of emotional cues such as micro or macro expressions or action units. Therefore, from the data point of view, each model, trained on a specific dataset, represents a recognition system of emotional expressions that is based on specific set of emotional cues, which once combined can lead to more accurate emotion recognition system. Consequently, one can expect that combining different region-specific cues together can reduce the inter-cultural bias in emotion recognition.

Multiple approaches have attempted to develop ensemble-like models for emotion recognition. For example, the model that achieves the state-of-the-art highest accuracy in the FER2013 dataset constructs an ensemble of 7 different CNNs^17^. Emotion prediction from the 7 CNNs are fused by a simple non-weighted sum average ensemble. A different approach was undertaken by^18^, where the authors use probability-based feature fusion. In addition, the maximum output across all CNNs can serve as a fusion function^19^. Finally, the work of^20^, proposes to concatenate features from 3 CNNs using a fully connected layer.

In the above mentioned ensemble-based approaches, each component algorithm use similar emotional cues. As a consequence there is no fusion of diverse emotional cues but rather similar cues learned from various data is being merged. Multi-modal approaches have also been studied as for instance in^21^ where the authors fused features from different modalities, such as videos and audios. However, the multi-modal fusion requires explicitly different physical modalities and does not explore in depth the different facial emotional cues.

In this paper we consider the problem of inter-cultural and inter-regional emotion recognition. When humans recognize emotional expressions they are able to adapt to various facial features, cultures and regional differences. However when artificial models are trained on region-specific datasets, the inter regional adaptation is not easily achieved by simple fine tuning. We postulate that each regional dataset for FER that is used for a model training represents a set of facial expression cues or modalities specific to the regional group contained in the dataset. We assume that these emotional cues are not completely compatible when applied to the FER of other regional groups. Rather we expect that each regional dataset can have a subset of emotional expressions captured by emotional cues that can be used in cross-regional emotional expression recognition. For instance, a set of cues used to accurately detect emotional expressions in one regional dataset might interfere with cues used to detect same emotional expression in another region-specific dataset. However, this set of cues can accurately detect different emotional expressions or subset of data samples of a different region-specific dataset. Thus while direct transfer learning or adaptation from one region-specific data set to another might not be successful, an adaptive combination of various emotional cues from different region-specific datasets could be designed.

We address the inter-regional facial emotional expression by empirical study on if and how an adaptively weighted combination of well known and pre-trained emotional cues can positively impact the inter regional FER recognition gap.

For this purpose, we propose a multi-cues attention model (MCAM) that combines region-specific pre-trained FER models (detector from now on) that we evaluate by simple combination, by fine-tuning each model individually and by ablation experiments. Each detector is exploiting a particular set of emotional cues, therefore by adaptively combining them we expect to minimize the inter-cultural emotional recognition specificity.

The emotional cues that we combine are action units, macro-expressions, micro-expressions and image-level features. The training and evaluation is performed over a set of selected datasets: JAFFE^15^, TFED^22^ (Chinese Individuals dataset), TFEID^23^ (Taiwanese Models dataset) and CK+^24^ (Caucasian and Afro-American dataset). Each of the emotional cues are obtained from detectors that are trained on a region-specific dataset and are then evaluated on datasets containing emotional expressions from various regions.

At first we evaluate an attention model trained over the outputs of three distinct emotional cues: action units, macro-emotions and micro expressions. In addition to these emotional cues we also add to the set of attention network inputs a set of image features. Action units are measures of facial muscle positions, macro expressions are labels describing discrete emotional expressions and micro-expressions are labels given to very small and often unconscious facial movements. Second we fine-tune each of the models to the evaluated dataset and we repeat the attention training. Finally, we evaluate combinations of emotional cues and for each combination we train the attention network.

As a results of these experiments, we found that for certain regional datasets fine-tuning of the individual emotional detectors is necessary in order to provide an improved emotion recognition. For TFED, TFEID and CK+, the proposed multi-cues attention model (MCAM) of region-specific pre-trained detectors is sufficient to improve the FER accuracy: attention trained over a target dataset is enough to improve the state-of-the-art accuracy on the same dataset. In contrast, for JAFFE, such combination and attention training is not sufficient: fine-tuning by any other similar region dataset improves the accuracy as long as image-level features were used.

A perfect evaluation accuracy (overfitting) was achieved for JAFFE dataset only when image-level features were used without any other emotional cues. For TFEID and CK+ perfect classification accuracy was obtained when image-level features were used independently of whether or not fine-tuning was used. On the contrary and independent of fine-tuning, for TFED the addition of image-level features strongly reduced the classification accuracy.

The results of this paper can be interpreted that learning is not always enough. In order to provide a good inter-regional emotion recognition, learned emotional cues sometimes have to be forgotten and replaced with others. Only then the model is able to fully recognize the regional specific emotional expressions.

This paper is organized as follows. Second section 2 presents related works and third section introduces the proposed model. Fourth section describes experiments and results. Fifth section discusses obtained results. Finally, sixth section concludes the paper.

## Background

There are numerous works on emotion recognition. Each of them has different approaches and often use different modalities. In^25^ the authors try to recognize emotions by speech signals, while authors in^26^ recognize emotions from text, and in^27^ the authors recognize emotions via brain signals. There are also approaches that combine different modalities resulting in multi-modal systems^21,28,29^. In this paper we focus only on emotion recognition from facial expressions. There are several distinct emotional cues used for image-based emotion recognition. In addition there are four super-categories of general emotional attribute descriptors that we consider as separate emotional cue.

**Macro-Expressions.** The most common approach used to classify emotions is to assign labels to a single facial expression. Approaches based on this model extract image-level features and directly map them to emotional labels. While the macro-emotions are not emotional attributes or cues but rather represent categories of facial expressions, an empirical mapping from images to categories is considered a specific type of holistic cue. For instance, using a deep neural network^14^ introduced the FER2013 dataset and an end-to-end learning approach for image level emotional labelling. Similarly, more recent approaches have exploited this approach such as in^30^. Current works are generally based on optimizing deep-learning models (especially convolutional neural networks). For example^31^, increased network depth by adding an inception-residual block^32^ replaced the last softmax layer with a linear SVM and optimized the loss from the L2-SVM (DLSVM) instead of the cross-entropy loss. Most common macro-expressions labels are: $\mathbb {M}_a=\{angry, scared, happy, sad, surprised, neutral\}$.

**Micro-expressions** are considered as an indicator of the true mental state of a person due to their spontaneous and subtle nature^33,34^. Micro-expressions are considered to be precursors of macro-expressions and they have been studied experimentally only in the last 10 years. There are two steps for micro-expression recognition. In the first step, spatial features are extracted from a set of video-frames. In the second step, temporal features are combined with the spatial features to form a micro-expression^35,36^. These two steps were recently combined in spatio-temporal features using 3D CNN^34^. The labels for micro-emotion recognition are $\mathbb {M}_i=\{positive, negative,surprised\}$ as well as an extended micro-emotion label-set $\mathbb {M}_{i+}=\{positive, negative,surprised, neutral\}$.

An **action unit (AU)** is a component of the Facial Action Coding System (FACS) that represents the movement of specific muscles of a face^1^. To recognize facial expressions belonging to a specific emotion, all active AUs are detected and then a combination of the detected AUs is classified into a specific emotion. Initial works used AU extraction models based on appearance (texture information of the image), geometry (shape properties of a face), and hybrid features^37^. However, newer models use deep learning methods^38–40^ for AU classification. There is a total of 64 AU specifying observable changes on the face obtained from^41^. From these we use eleven AUs that are used in standard emotional recognition approaches $\mathbb {M}_u=\{1,2,4,5,6,7,12,15,20,23,26\}$. For example, if a face in an image is detected to have AUs 6 and 12 active, it indicates that the person is expressing happiness^30^.

**Image Features** are extracted as image agnostic descriptors using a pre-trained convolutional neural network. The image features can be extracted through a variety of existing networks or a specific network can be trained specifically on a target dataset^42^. Usually, the image features resulting from an input image are further used for classification and are considered to be one of the most descriptive. Image features are also one of the most common approaches when generating Macro-expressions (cf. above). While these features are not completely content agnostic, their descriptive power has been shown to be almost universal^43^. Therefore we use the image features as general descriptors and we intend to use them to fill the gap in recognition that other emotional specific markers are not able to cover. The features are extracted using a VGG11 model, pre trained on ImageNet.

## Proposed method

**Figure 1:**
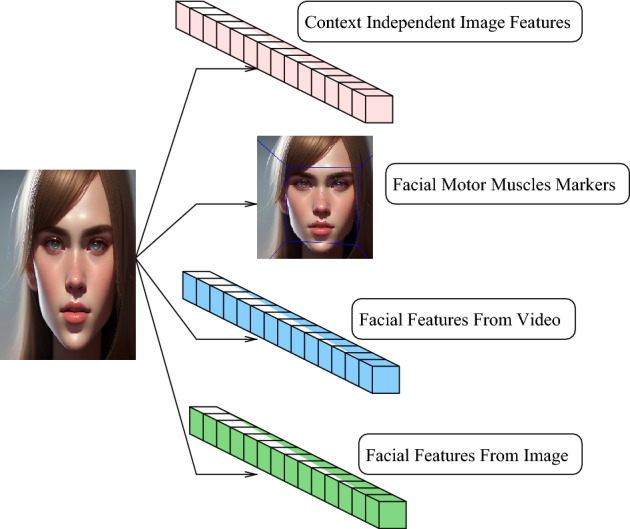
The four kinds of features used in our proposed facial emotional expression detector.

The principle behind the proposed methodology is to determine if the existing detectors of emotional cues can be combined in order to bridge data bias and regional bias. For this purpose we consider four different emotional cues, each detecting a different aspect of the facial emotional expression. In particular we use a context-independent set of features, a set of physiological markers, short term, involuntary and low intensity facial expression features and finally a set of macro-emotional labels. The overall schema of the used components for the facial attributes/emotional cues is shown in Fig. 1.

Going bottom-up, the the image features $\mathbb {F}$ are considered, region, task and context-independent low-level features. These features were obtained as a result of training on a general dataset that contains a variety of objects, animals and humans. They are expected to be less biased towards any regional data. We selected these features in order to get a feature set as diverse as possible; a feature set independent from the target topic can capture facial attributes that more specialized feature sets can repress due to their low statistical significance.

The second from the bottom are the Action Units $\mathbb {U}$. Mapping of muscular motion through observable facial changes provides a set of coordinates of facial markers that we consider emotion and individual expression independent. While the AU are face-specific markers, we consider them to be region-independent because the muscle activity measurement is a face-specific feature that occurs in any human and is not related to emotional expression only. The action units are used to calculate individual emotional expressions by considering only a subset of facial muscles because the motion of all facial muscles is not necessary to determine muscles’ spatial offset characterizing an emotional expression. Therefore the AU can be seen as specific facial-like features without being biased towards any facial type or social behavior.

Third are the features obtained from video recording of emotional expressions $\mathbb {I}$. Micro-expressions are considered to be involuntary, small magnitude facial expressions occurring in time. By definition micro-expressions are simpler (less various), have smaller magnitude and can occur spontaneously or as a precursor to full macro-emotional expressions. Therefore, we use the micro-expressions in order to detect emotional expressions of smaller magnitude and variety. In addition, the micro-expression detector is trained on short video sequences: the principle when classifying images with a sequence detector is to classify facial features that match a specific frame index in a sequential emotional expression. Therefore the resulting classification contains information on trends of how this emotional expression image evolves over time. Also, because the micro-expressions are not full fledged emotional expressions, we consider the features extracted from the image to be mapped to only a sub-set of macro-emotional labels. Finally, because the intensity of micro-expressions is in general quite low, we consider the result of micro-expression detection complementary to the detection of other emotions and facial expressions.

The last facial attributes are the macro expressions $\mathbb {A}$. The macro expressions represent the standard facial expression labels such as happy, angry or sad. Unlike the other emotional cues, the macro expressions while being a set of emotional cues they are also the target of facial emotional expression detection: in our experiments the final target is the classification into the macro-expression labels. The macro-expressions are however also detected by a dedicated detector and the result of the detection is similar to all the other emotional cues. The features used for determining the macro-emotions are obtained from training on an image dataset of facial emotional expressions. As such they are very specific to the human face, to emotional expressions and should be the most sensitive to regional bias.

**Table 1:** The detectors of emotional cues used in our approach.

Feature	Detector	Mapping	Classifier
$\mathbb {F}$	$D_f$	$I\rightarrow \mathbb {M}_f$	N
$\mathbb {U}$	$D_u$	$I\rightarrow \mathbb {M}_u$	N
$\mathbb {I}$	$D_i$	$I\rightarrow \mathbb {M}_i$	Y
$\mathbb {A}$	$D_a$	$I\rightarrow \mathbb {M}_a$	Y

Significant values are in bold.

For most of the emotional cues used we define a classifier. Each classifier takes as input a specific emotional cue and outputs a label of recognized emotional expression. The features, detectors, mappings and the presence of a classifier of all used algorithms are shown in Table 1. The column of Table 1 shows the features extracted from the input data, the second column shows the detector that provides this information and the third column shows the mapping of the detector from the input image to the emotional cues. The fourth column indicates if in the default usage a classifier is used to generate the emotional cues. For instance in the case of image features $\mathbb {F}$ the emotional cues $\mathbb {M}_f$ are equivalent to $\mathbb {F}$ (the change of nomenclature is introduced for a unified representation). However the facial features from image $\mathbb {A}$ are mapped to emotional label $\mathbb {M}_a$ by a classifier.

Our method starts by first taking an image (frames), process it with the different emotion cue detectors from Table 1. The emotional cues from all detectors are combined and are classified into a label $l\in \mathbb {M}_a$. The classification is performed using an attention module that predicts the emotional label.

**Figure 2:**
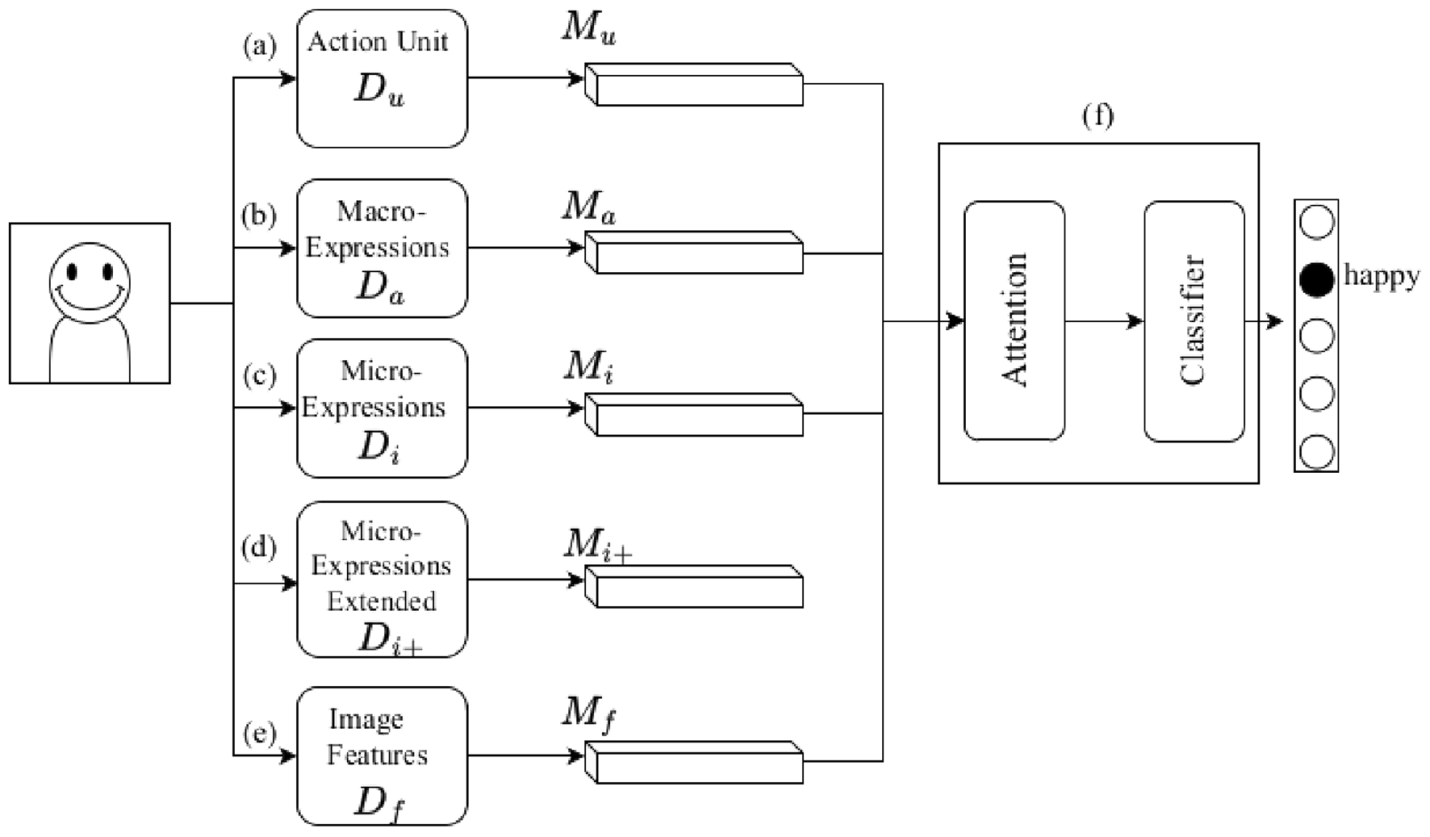
Overview of the proposed MCAM framework.

The architecture of the proposed multi cues emotion model MCAM is illustrated in Fig. 2. We use an action units detector (Fig. 2a), a macro-expression detector (Fig. 2b), a micro-expression detector (Fig. 2c and d), and image features extractor (Fig. 2e). We combine them using an attention-based network (Fig. 2f).

### Models

Each of the models used in this work is a well-known algorithm. The MCAM attention network $\mathcal {A}$ is a multi-layered perceptron. It contains three hidden layers, each with ten neurons. The size of input layer varies according to the configuration. The rectified linear unit is used as an activation function. The learning rate is 0.001. The image features detector $D_f$ is a pre-trained VGG11 CNN which outputs the image features $\mathbb {M}_f=D_f(I)$. The output vector of $D_f$ contain 2752 values. The AU were obtained from $D_u$ that uses OpenFace 2.0 toolbox. The $\mathbb {M}_u$ are extracted from the detected face and are classified using an SVM^41^. Intensities of 11 AUs that are responsible for 6 basic emotions were used in our model. Micro-expressions cues $\mathbb {M}_i$ and $\mathbb {M}_{i+}$ are obtained from a 3D CNN trained on a sequence of images taken in a very short time span (trained on SMIC and SMIC+ respectively). We use single image as a sequence to obtain an emotional label $\mathbb {M}_i=D_i(I)$ from the 3D CNN trained and evaluated on sequence of different images. In addition, as the 3D CNN trained on the SMIC dataset showed low accuracies for datasets FER13 and CK+, and the facial expression datasets contained a neutral face, the 3D CNN was fine-tuned with the CK+ dataset^24^ and re-classified for four facial expressions $\mathbb {M}_{i+}=\{positive, negative,surprised, neutral\}$. This detector is referred to as $\mathbb {M}_{i+}=D_{i+}(I)$. In addition $\mathbb {M}_i$ and $\mathbb {M}_{i+}$ were evaluated on agglomerated categories: $\{angry, scared, sad,\} \rightarrow \{negative\}$, $\{happy\}\rightarrow \{positive\}$, $\{surprised\} \rightarrow \{surprised\}$ and $\{neutral\}\rightarrow \{neutral\}$ was used only for $\mathbb {M}_{i+}$.

The macro expressions $\mathbb {M}_a$ were obtained from a $D_a$ based on the model proposed in^42^. It performs the mapping $\mathbb {M}_a = D_a(I)$. The result contains labels from $\text {angry, scared, happy, sad, surprised, neutral}$.

### The attention network

The attention network assigns weights to each of its inputs in order to strengthen the impact of different input modalities. While the attention computational mechanism is well known we follow the approaches of some of the more recent works^44,45^. The concept of the model computational attention is that successive attention models are able to extract the most important verbal tokens and provide the state-of-the-art accuracy in the translation tasks. We use the concept of attention by combining the emotional cues and image features and train the attention network for the final classification. In particular our model computes the soft weight for each of the possible emotional labels. The method is therefore an instance of soft algorithm selection where the output of the attention network is the confidence score distributed over each of the available target emotions.

All together the cues from $\mathbb {M}_u$, $\mathbb {M}_a$, $\mathbb {M}_{i}$, $\mathbb {M}_{i+}$ and $\mathbb {M}_f$ are used as input to the attention multi-layer perceptron (Fig. 2f). We define $\mathbb {S} =\{\mathbb {M}_a, \mathbb {M}_i,\mathbb { M}_{i+}, \mathbb {M}_u, \mathbb {M}_f \}$ as the set of all emotional cues. We will refer to a subset of $\mathbb {S}$ as follows. A subset $\mathbb {S}_{-i}$ refers to the emotional cues that are missing from the subset, therefore in this case $\mathbb {S}_{-i}=\{\mathbb {M}_a, \mathbb {M}_{i+}, \mathbb {M}_u, \mathbb {M}_f \}$. We also define that a subset $\mathbb {S}_{f,i}$ contains only the $\{\mathbb {M}_f, \mathbb {M}_i\}$ emotional cues. The introduction of this positive and negative notation is convenient for indicating the components of the original set $\mathbb {S}$ more concisely.

The MCAM attention model represents a class of parametric models specified in terms of the input emotional cues. In order to represent the various configuration we will use $\mathcal {A}$ subscripted by the indices of used emotional cues set. The default model $\mathcal {A}$ is used to represent the MCAM model that uses all studied emotional cues defined in the $\mathbb {S}$ set. An instance specified by $\mathcal {A}_{-i+,-u}$ indicates an attention model that takes as input the set $\mathbb {S}_{-i+,-u} = \mathbb {S}_{a,i,f}=\{\mathbb {M}_a, \mathbb {M}_i, \mathbb {M}_f \}$.

### Training

The combination of the different emotional cues is tested on a series of cross-dataset evaluation tests: we train the emotional cues on one region-specific dataset and evaluate them on a different region-specific dataset.

Specifically, we combine the regionally pre-trained, region-agnostic and task-agnostic detectors to determine how the region-specific methods can be manipulated into a universal, region-independent emotional expression detectors. As the used emotional cues range from holistic emotional expressions labels to low-level image features, the proposed methodology can be described on two different levels. First we analyze how micro and macro-expression detectors, trained on regional data, are affected by the regional specificity. This means that we consider combinations of regionally trained facial expression detectors and determine how they generalize to other region-biased expressions for which they have not been trained. Second, we study how image features and the action units can help to regionally-debias the regionally trained emotional expression detectors. While the image features are used in the macro and micro emotions detectors they are subject to bias due to them being used as an input to a region-specific classifier. Using these features as-is is therefore a method to bridge a possible gap in representation of the facial information that might be lost during the learning.

Three sets of experiments are used to determine the emotional cues interference and bias. At first, we use pre-trained emotional cues detectors and learn the attention using an MLP. This experiment is then extended into learning attention over individual detectors: we learn the attention using a single detector at a time. The last approach is focused on fine-tuning the individual detectors and then learning the attention.

### Global and local adaptation

We distinguish two types of learning approaches: globally and locally adapted features. In the global adaptation mode (GAM) we train a network initiated from scratch on the target dataset while in the local adaptation mode (LAM) we only fine-tune a model already trained on a different dataset. For instance, in GAM mode, to classify the JAFFE data, we train the attention module on the train set of JAFFE and evaluate the MCAM on the eval subset. In the LAM mode, we first fine-tune the existing emotional cues on the training dataset of the target regional data. Then we train the attention module on the same training set and evaluate the whole MCAM on the eval dataset.

The image features detector $D_f$ and the action units detector $D_u$ are not learned or fine-tuned but are instead used pre-trained and out-of-the box. The macro emotional expression detector $D_a$ was a VGGnet based CNN (macro emotional labels), pre-trained on FER2013. The micro and micro+ emotional expression detectors $D_i$ and $D_{i+}$ respectively, were both trained on SMIC. The main difference between $D_i$ and $D_{i+}$ is that $D_{i+}$ was also fine tuned on CK+.

The two approaches GAM and LAM thus result in detectors that are either trained on FER2013 and SMIC with the attention network trained on the target dataset or in detectors fine-tuned on the target dataset with the attention trained on the same target dataset.

for additional details on models, training and testing procedures please consult the appendices.

## Experiments and results

### Datasets

For training the MCAM model, we separate data into train and test splits with 80% and 20% ratios. In total we consider five datasets: FER2013, SMIC, JAFFE, TFED and TFEID. The two datasets FER2013 and SMIC were used only for the training the macro-emotions detector $D_a$ and micro-expression detectors $D_i$/$D_{i+}$.

### Accuracy of the baseline models

In this section we provide experimental results for the state-of-the-art methods, $\mathcal {A}$, $\mathcal {A}_{-f}$ and the individual $D_i$, $D_{i+}$ and $D_a$ emotional-cue detectors. The general concept of these experiments is to simply verify the accuracy of the detectors that provide the emotional expressions labels as output by default.

**Table 2:** Accuracies in percentage for the four datasets used for testing MCAM approach.

Dataset	SOTA	$\mathcal {A}_{-f}$	$\mathcal {A}$	$D_a$	$D_i$	$D_{i+}$
Jaffe	96^46^	95	95	54	47	49
TFEID	96^46^	**98**	**97.22**	72	44	15
TFED	79^22^	**92.44**	**86.36**	65	33	30
CK+	99^47^	**99**	98.63	85	59	90

Significant values are in bold.

The summary of the results is shown in Table 2. The last three columns show the accuracy of macro emotion recognition using directly ${D}_a$, $D_i$ and $D_{i+}$ . The individual detectors $D_a$, $D_i$ and $D_{i+}$ are not trained on datasets that were used for testing, except the $D_{i+}$ (fine tuned on CK+). The MCAM models $A_{-f}$ and *A* were LAM trained on each of the datasets respectively. We do not report the accuracy for the action units and image features because $D_u$ and $D_f$ do not provide labels for emotional expressions by default. The second column provides the state-of-the-art accuracy for the given datasets, while the third and fourth columns show our method’s results for $A_{-f}$ and *A*, respectively. The values in each column indicate the accuracy on the test set: the testing of the learned model was performed on 20% of each used dataset. Cells colored in blue show where our method matched or improved the result of the SOTA method (second column).

The first observation is that the accuracy of the single emotional modality detectors (columns five, six and seven in Table 2) is much worse and always worse than when they are used together in the studied MCAM based approaches. It can be seen that on all three of the datasets (JAFFE, TFED, TFEID) all three detectors performed by at least 15% of accuracy below the SOTA. Also $D_i$ performed poorly on CK+ which is not regionally different from the training set of $D_i$ and $D_{i+}$, SMIC. Interestingly $D_a$ performed best on CK+ as well as did $D_{i+}$. $D_{i+}$ was LAM trained on CK+ and therefore it is expected that the results would be acceptable. However, $D_a$ was only trained on FER2013 and still the accuracy on CK+ was acceptable. We observe that the detectors $D_a$, $D_i$ and $D_{i+}$ have show performance degradation with respect to regionally biased datasets. However the MCAM model showed very steady improvement using only LAM training. This means that while the information for classification of emotional expressions from different regions is available in the emotional cue detectors it is not properly used for classification on a dataset-by-dataset basis.

The second observation is that for CK+, TFED and TFEID, our proposed method was able to outperform the current SOTA approaches without being fine-tuned. Also note that in the case of JAFFE the classification using MCAM increases the result by more than 40% in accuracy when compared with the highest accuracy of emotional detector used (here $D_{a}$ for JAFFE ). For TFEID the increase is more than 25%, for CK+ it is 8% and for TFED the increase is up to 20%.

Observe that the MCAM $\mathcal {A}_{-f}$ outperformed the $\mathcal {A}$ model: removing the image features increased the accuracy. Because the training data was large enough we consider that this difference is not due to the lack of training samples. Rather we cautiously conclude that the $M_f$ features might be causing interference in the classification process.

In addition, one can stipulate that even though the base models were not trained on any of the test sets under evaluation, and individual emotional detectors have a quite low accuracy, together (with AU emotional cues) they provide enough information to extract the most salient emotional expressions. One could argue that the training of the MCAM models is in a sense similar to a learning transfer on each of the individual component emotional recognition models. While such an assumption is valid, our goal here is different: instead of adapting each model to a particular dataset, we are interested as to whether the results of the originally trained models can be simply re-weighted such that decisions on new datasets can be obtained from a mixture of individual models’ positive and negative results. This approach can be thus classified as a sensitivity and discrimination testing of existing methods.

Finally, our initial hypothesis that using macro expressions, action units, micro-expressions and/or $\mathbb {M}_f$ features would allow us to bridge the gap of emotional expression in **all** of the selected datasets was not fully achieved: our method did not outperform the SOTA for the JAFFE dataset.

### Single cue classifiers

**Table 3:** Evaluation of MCAM models with individual emotional cues.

Dataset	LAM					GAM	
	$\mathcal {A}_{a}$	$\mathcal {A}_{i}$	$\mathcal {A}_{i+}$	$\mathcal {A}_{u}$	$\mathcal {A}_{f}$	$\mathcal {A}_{a}*$	$\mathcal {A}_{i}*$
JAFFE	72.97	36.11	27.03	70.24	**100**	83.78	45.95
TFEID	77.78	50.00	60.87	69.68	**98.19**	86.966	62.22
TFED	87.12	26.52	28.79	79.54	75.19	88.64	32.58
CK+	88.62	37.72	48.81	63.40	**100**	96.41	47.02

Significant values are in bold.

In order to understand the results from the composition of emotional cues, we decided to analyze MCAM models that use single emotional detectors. For this purpose we took every individual detector and used its outputs as inputs to the attention network $\mathcal {A}$. The attention network was then LAM trained to the dataset under evaluation. In addition we also performed two experiments with GAM training using the $D_a$ and $D_i$.

Table 3 shows the summary of the experiments. The first column shows the dataset under evaluation, columns two to six show the different LAM trained single detector MCAM models and the last two columns show the GAM trained $\mathcal {A}_a$ and $\mathcal {A}_i$.

Unexpectedly, two of the classification tasks obtained perfect (overfitted) results. In particular the JAFFE and the CK+ dataset was perfectly classified when the $\mathcal {A}_{f}$ MCAM model was used. was used In addition very high accuracy of classification was also achieved for the TFEID dataset with the $\mathcal {A}_{f}$ model. The interesting result is that while JAFFE, TFEID and CK+ have their accuracies increased over the SOTA results from Table 2 the results for TFED show a decrease in accuracy. In addition the result of $\mathbb {A}_{a}$ and $\mathbb {A}_{i}$ GAM trained outperform their LAM trained counter parts.

### GAM

Next, to verify if better performance could be achieved by re-weighting the results of the emotional detectors we performed a set of GAM training experiments. For this purpose the ${D}_{a}$ and the ${D}_{i}$/${D}_{i+}$ models were fine-tuned on the datasets used for evaluation and then cross-evaluated against the MCAM model $\mathcal {A}_{-f}$. Table 4 shows the datasets used for the GAM training as rows and the datasets used for evaluation as columns. The first observation is that GAM learning is not always a symmetric process. For instance, the model GAM trained with JAFFE has much better results on TFED than the model GAM trained with TFED on JAFFE. The improvement from GAM training is observed in a symmetric manner for TFED/TFEID, CK+/TFEID, JAFFE/TFEID and CK+/JAFFE. Moreover, with the refining of the feature detectors the accuracy on the JAFFE dataset reached, but did not improve beyond the SOTA performance.

**Table 4:** Accuracy of fine-tuned $\mathcal {A}_{-f}$ on base models in percentage.

	Datasets	Evaluated on			
		JAFFE	TFEID	TFED	CK+
Fine	JAFFE	**96**	78.70	**94.21**	78.94
-tuned	TFEID	91.30	**100**	**95.65**	91.30
with	TFED	84.85	90.91	**95.45**	89.39
	CK+	92.86	90.48	**94.05**	98.80

Significant values are in bold.

Therefore we evaluated the GAM trained $\mathcal {A}$ (Table 5). The first interesting observation is that the addition of the image features extracted from using ${D}_{f}$ increased the performance of all models in a non-linear manner. Interestingly, the GAM training from any of the datasets resulted in 100% accuracy when evaluating the accuracy on CK+ and TFEID. For JAFFE, all GAm training using datasets beside CK+ resulted in an increase in accuracy. However, for TFED the GAM training did not result in maximum accuracy when compared to the results from Table 4 and Table 3, but the accuracy is increased with respect to the SOTA (Table 2).

**Table 5:** Accuracy of fine-tuned $\mathcal {A}$ on base models in percentage.

	Datasets	Evaluated on			
		JAFFE	TFEID	TFED	CK+
Fine	JAFFE	**97.5**	**100**	80.30	**100**
-tuned	TFEID	**97.23**	**100**	83.33	**100**
with	TFED	**97.14**	**100**	83.37	**100**
	CK+	94.44	**100**	81.82	**100**

Significant values are in bold.

Also observe, that for JAFFE 100% accuracy was not achieved as is the case when only $\mathcal {A}_f$ (Table 3). This could further confirm our initial observation that there is a general interference between the learned macro-emotions, micro-expressions and action units and the image features. While the combination and the GAM training works well for TFEID and CK+, its effect is still positive (improving over the SOTA) on the JAFFE dataset but it reduces the accuracy for TFED (for $\mathcal {A}$). This is very interesting because the simple operation of adding $\mathbb {M}_{f}$ features acts on one hand as improving the emotion detection due to pixel level information and on the other hand as pixel level noise reducing the accuracy of classification. Interestingly TFED had the highest accuracy with single emotional detectors given by the $D_u$ and by the ${D}_{a}$ (Table 3). However it also had one of the lowest classification accuracies when only the $\mathcal {A}_{i}$/$\mathcal {A}_{i+}$ models were used: it can be assumed that the finer grain features or cues are be interfering with the more distinctive emotional cues and therefore using too much fine-grain information is diminishing the performance on the TFED dataset.

### GAM vs LAM

**Figure 3:**
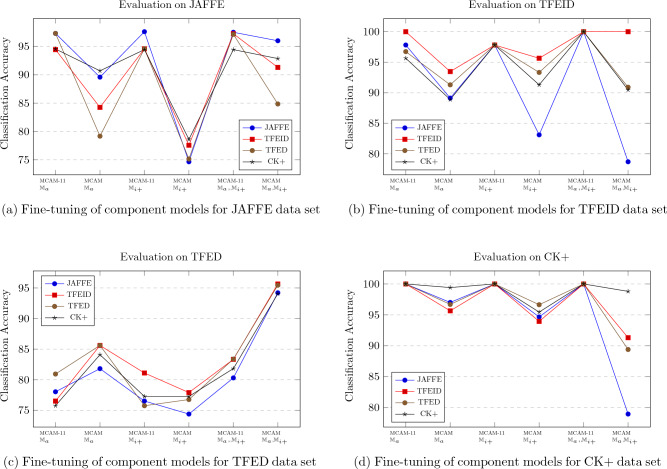
Ablation experiments on all four datasets evaluation samples.

The final set of experiments was performed in order to better understand the effect of the GAM training on the individual datasets. For this purpose a set of ablation experiments were conducted. The experiments evaluate the accuracy of the $\mathcal {A}_{-i}$ and $\mathcal {A}$ models that are component-wise GAM trained and evaluated on the same dataset. Figures 3a to d show the GAM training ablation study for each of the datasets. Each graph shows the GAM training using all four datasets (one line per fine-tuning dataset) and each data point shows GAM trained models. The first, third and fifth data points show the $\mathcal {A}$ with different components algorithm GAM trained while the second, fourth and sixth data point shows the $\mathcal {A}_{-f}$ under same GAM trained conditions. Similarly to the previous experiments, the only GAM trained component models are $D_a$ and $D_{i+}$.

Figure 3a shows the evaluation on JAFFE. The main observation is that JAFFE is most sensitive to the addition of the $\mathbb {M}_{f}$ pre-trained image features, therefore confirming the previous observations. In addition, when $\mathbb {M}_{f}$ features are not used the GAM training that works the best is not self GAM training (JAFFE training, JAFFE evaluating) but rather the CK+ GAM trainining (black line, second datapoint). Finally, as can also be seen in Table 5 (column JAFFE), when the $\mathbb {M}_{a}$ and $\mathbb {M}_{i+}$ algorithms are GAM trained and are used in $\mathcal {A}$, the GAM training on JAFFE, TFED and TFEID results in the same classification accuracy.

A similar trend can be observed for the evaluation of the ablation on the TFEID dataset (Fig. 3b). The main difference is that in this case, the self-tuning (TFEID tuning, TFEID evaluation) results always in highest accuracy independently on the ablation model evaluated, while the fine tuning using JAFFE in general results in the lowest or one of the lowest obtained accuracy. In addition, when only the $\mathbb {M}_{i+}$ or the $\mathbb {M}_{a}$ and $\mathbb {M}_{i+}$ are fine-tuned, each shows the same accuracy improvement (data point three and five).

In the case of TFED (Fig. 3c) the results are different from the two previous cases. The first observation is that the best result is obtained only when all components of emotional detectors have been GAM trained (sixth data point). In addition, unlike in the previous two ablation cases, the best result is when the $\mathcal {A}_{-f}$ (not $\mathcal {A}$) is used with all components detectors GAM trained. The second observation is that the self GAM training (GAM trained on TFED, evaluated on TFED) has resulted in very similar accuracy improvement profile as the GAM training on TFEID. The only considerable different case is the first data point, where only ${D}_{a}$ has been GAM trained and is used in $\mathcal {A}_a$. In addition when only the ${D}_{i+}$ was GAM trained, the best GAM training is the one performed on TFEID and not the self GAM training.

The final case is the evaluation of the CK+ dataset (Fig. 3d). The accuracy changes due to GAM training of individual component detectors is similar to the cases of JAFFE and TFEID: the addition of $\mathbb {M}_{f}$ features results in observably increased accuracy of classification. The main difference is that in average the accuracy on the CK+ dataset is higher than on any other evaluated dataset. The reason for this difference is most probably due to the fact that both the ${D}_{a}$ and ${D}_{i+}$ have been trained on FER13 and SMIC respectively. Both these datasets are closer in the data bias to CK+ than to any other evaluated dataset. FER13 and SMIC datasets contain mostly Caucasian and African-American individuals with few Asian individuals. In addition the ${D}_{i+}$ was GAM trained on CK+ .

In addition, while self GAM training also generates in general the highest obtained accuracy, when only the ${D}_{i+}$ is GAM training, the highest accuracy is given by the TFED GAM training and not self GAM training by CK+.

## Discussion

The most important observation made in this work is the excluding nature of certain features. The $\mathbb {M}_{f}$ image level features improved two (CK+, TFEID) out of the four datasets up to 100% accuracy. In addition $\mathbb {M}_{f}$ image features also allowed us to attain 100% for JAFFE but only if no other emotional cues were used. This effect empirically indicates that for JAFFE the $\mathbb {M}_{f}$ features interfere with all other emotional cues trained on other regional datasets. Such interference is also observable on TFED where $\mathbb {M}_{f}$ features actually reduce the accuracy of recognition.

Combining these observations can lead to a model for emotional recognition: in order to recognize emotional expressions for various regional groups, certain emotional cues used to identify emotions in some regional group are preventing the efficient recognition of emotion in another regional group. This would indicate that the emotion recognition bias is not due to lack of effective emotional cues but rather due to the lack of effective forgetting of pre-learned emotional cues. For instance in JAFFE only the fine-grain image-level features seem to be effective. This would indicate that emotional expression in JAFFE are the least expressive. On the other hand the emotional expression recognition accuracy in TFED is reduced by introducing the image-level features: this would indicate that emotions in TFED are closer to the pre-learned model on FER2013 than is JAFFE.

Finally one can summarize the expressed emotions to a relational graph. TFEID and CK+ represent emotional models that are effectively described by all emotional cues, JAFFE is exclusive to only image-level features and TFED is exclusive to explicitly not use image-level features.

The perfect accuracy of classification on the various test sets, shows a possibility of overfitting. Therefore the results provided in this paper can be seen as an approximate evaluation. The datasets did not have the same number of images and there were only six output categories. With a relatively small number of images the classifier might not have enough of parameters or has too many (and thus memorizes) to properly learn the image. In addition the empirical experiments in this work showed bias between different regional based datasets. However we did not mix datasets and we did not analyze them individually. Therefore the origin of the bias can be the data content (different regional groups), collection method (local, random, etc), data set quality (noise, resolution, etc) or the lack of diversity within the collected data.

While the overfitting of the classification is a negative result, it shows which emotional cues and image features are necessary to perfectly embed or memorize the emotional expressions. Interestingly the overfitting happens under different combinations of emotional cues for the different datasets. Looking at the results, the most overfitting occurs when the VGG-11 features are used. This would indicate that the number of the features is enough to embed the whole dataset entirely. However because this trend is not absolute we also detected some interference between features and emotional cues.

## Conclusion

In this paper we empirically showed that there is an observable bias when one attempts to generalize the current SOTA emotion recognition methodology. We were able to bridge the bias between some datasets but we also showed that some datasets are not directly compatible: for high accuracy emotion recognition they use mutually interfering features. In addition, the distinctive and occasionally contradictory nature of regionally-specific emotional expression can serve to obstruct and confound the transfer of learning of emotional recognition based on the facial expressions of one region to that of another, thus preventing the achievement of a “perfect” classifier. As we determined, it is sometimes better to “forget” what was previously learned, and proceed from first-principles. In such cases, it is our hypothesis that a fusion-scoring approach across multiple categories may nevertheless partially overcome this obstacle to improve (but not perfect) the emotion recognition outcomes.

As a future work we plan to investigate the weight of individual emotional cues in emotion detection in different ethnic datasets and develop a generative model for emotion expression generation.

### Supplementary Information

Supplementary Information.

## Data Availability

Each of the used datasets can be found on their respective links as shown below: TFED: For the dataset, the authors of the original paper introducing the public version of the dataset at this address https://journals.plos.org/plosone/article?id=10.1371/journal.pone.0231304 should be contacted. TFEID: For the dataset, the authors of the original paper introducing the public version of the dataset (at this link http://www.psy.ntu.edu.tw/vnl/paper/Chen_2009_Exodatabase_SPIE.pdf) should be contacted. JAFFE: https://zenodo.org/record/3451524. CK+: https://www.kaggle.com/datasets/shawon10/ckplus. FER2013: https://www.kaggle.com/datasets/msambare/fer2013. SMIC: https://www.oulu.fi/en/university/faculties-and-units/faculty-information-technology-and-electrical-engineering/center-machine-vision-and-signal-analysis.
