# Author Correction: Study on emotion recognition bias in different regional groups

**DOI:** 10.1038/s41598-023-36806-w

**Published:** 2023-06-19

**Authors:** Martin Lukac, Gulnaz Zhambulova, Kamila Abdiyeva, Michael Lewis

**Affiliations:** Department of Computer Science, Nazarbayev University, Kabanbay Batyr 53, Astana, 010000 Kazakhstan

Correction to: *Scientific Reports* https://doi.org/10.1038/s41598-023-34932-z, published online 24 May 2023

The original version of this Article contained an error in the Data availability section.

“All the datasets (FER, SMIC, SMIC+, TFED, TFEID, JAFFE) used and/or analysed during the current study available from the corresponding author on reasonable request.”

now reads:

“All the datasets (FER, SMIC, SMIC+, TFED, TFEID) used and/or analysed during the current study available from the corresponding author on reasonable request.”

The original Article has been corrected.

