# Author Correction: Study on emotion recognition bias in different regional groups

**DOI:** 10.1038/s41598-023-40126-4

**Published:** 2023-08-10

**Authors:** Martin Lukac, Gulnaz Zhambulova, Kamila Abdiyeva, Michael Lewis

**Affiliations:** Department of Computer Science, Nazarbayev University, Kabanbay Batyr 53, Astana, 010000 Kazakhstan

Correction to: *Scientific Reports* 10.1038/s41598-023-34932-z, published online 24 May 2023

The Data availability section in the original version of this Article was incorrect. It now reads:

“Each of the used datasets can be found on their respective links as shown below:

TFED: For the dataset, the authors of the original paper introducing the public version of the dataset at this address https://journals.plos.org/plosone/article?id=10.1371/journal.pone.0231304 should be contacted.

TFEID: For the dataset, the authors of the original paper introducing the public version of the dataset (at this link http://www.psy.ntu.edu.tw/vnl/paper/Chen_2009_Exodatabase_SPIE.pdf) should be contacted.

JAFFE: https://zenodo.org/record/3451524

CK+: https://www.kaggle.com/datasets/shawon10/ckplus

FER2013: https://www.kaggle.com/datasets/msambare/fer2013

SMIC: https://www.oulu.fi/en/university/faculties-and-units/faculty-information-technology-and-electrical-engineering/center-machine-vision-and-signal-analysis”.

The original Article has been corrected.

