# Supplementary material for: Study on emotion recognition bias in different regional groups
Source: Sci Rep. 2023 May 24;13:8414. doi: 10.1038/s41598-023-34932-z (PMC10209154; doi:10.1038/s41598-023-34932-z)
Supplement: Supplementary file 1 — Supplementary Information. [file 41598_2023_34932_MOESM1_ESM.pdf]

Study on Emotion Recognition Bias in Different Regional Groups

Martin Lukac[1*†], Gulnaz Zhambulova[1†], Kamila Abdiyeva[1] and Michael Lewis[1]

[1*]Department of Computer Science, Nazarbayev University, Kabanbay Batyr 53, Astana, 010000, Kazakhstan.

*Corresponding author(s). E-mail(s): martin.lukac@nu.edu.kz; Contributing authors: gulnaz.zhambulova@nu.edu.kz; kabdiyeva@nu.edu.kz; mlewis@nu.edu.kz;
[†]These authors contributed equally to this work.

Keywords: Facial Expressions Classification, Emotion Recognition, Regional Bias

Appendix A Training Parameters

A.1 Attention Network

The inputs to the attention network are the combined outputs of the different detectors:

A.1.1 Input-Output Mapping

- 6 inputs from the macro-emotional expression detector VGGnet (probability distribution of 6 basic emotional labels $\mathbb{L}$)
- 3 inputs from the 3D-CNN detecting the micro-expressions(probability distribution of 3 emotional labels: positive, negative, surprised)
- 4 inputs from the 3D-CNN+ fine-tuned (probability distribution of 4 emotional labels: positive, negative, surprised, neutral)
- 11 inputs from Action Units (1, 2, 4, 5, 6, 7, 12, 15, 20, 23, 26) - numbers represent intensity of a specific action unit (regression model used)
- If the outputs from the feature image detector VGG11 are used, there is additional 2752 points (image features) that are added to the input

A.1.2 Data Preprocessing

- 15% of data was used for testing and 85% for training.
- Features extracted form the input images are standardized by removing the mean and scaling to unit variance.

A.1.3 Attention MLP Architecture

- the default attention architecture is a MLP with 3 hidden layers, 10 neurons in each layer. If the VGG11 image features are used as input, the MLP is constructed with 3 hidden layers, 50 neurons in each layer (neuron number is increased as input size increased)
- The rectified linear unit function as an activation function for the hidden layers.
- Adam, a stochastic gradient-based optimizer, is the solver for weight optimization.
- Learning rate is 0.001.
- Maximum number of iterations = 2000.
- The output contains 6 neurons (probability distribution of 6 basic emotions)

A.1.4 Accuracy Measure

Macro average (averaging the unweighted mean per label) precision is taken as an accuracy indicator. Precision is the ratio $\frac{tp}{(tp+tn)}$ where tp is the number of true positives and fp the number of false positives.

A.2 Classifier for Single Detectors

When each of the model is evaluated individually, we use a classifier to obtain the final result. The classifier is always a Multi-Layered Perceptron and depending on the case it has the following parameters:

- For VGGnet evaluation: 1 layer with 10 neurons. Input: probability distribution for 6 emotions from VGGnet.
- For 3D CNN evaluation: 1 layer with 10 neurons. Input: probability distribution for 3 emotions from 3D CNN.
- For AU evaluation: 1 layer with 10 neurons. Input: 11 points from Action Units (1, 2, 4, 5, 6, 7, 12, 15, 20, 23, 26) - numbers represent intensity of a specific action unit (regression model used).
- For VGG11 evaluation: 3 layers with 50 neurons on each layer. Input: 2752 image features 15% of data for testing and 85% for training.
- No normalization is used.

Appendix B Emotional Cues Detectors

B.1 Emotional Expression from Image Detector

We decided to use VGG9 due to the ratio of performance and computational cost. This decision was made after the comparison of various VGG like models. The comparison is summarized in Table B1.

Model Name	Reported Accuracy	Architecture	Source
VGG-9	66.96% (75 epochs)	3 Conv, 3 FC	
VGG-10	72.7% (300 epochs)	8 Conv, 2 FC	[1]
VGG-11	73.28% (300 epochs)	8 Conv, 3 FC	[2]
VGG-16	69.14% (30 epochs)	13 Conv, 3 FC	[3]
VGG-16+GAP	65.03% (30 epochs)	13 Conv, 3 FC	[4]
VGG-19	68.8% (30 epochs)	16 Conv, 3 FC	[4]

Table B1 Comparison of the various VGG architectures when trained for facial emotion recognition

B.1.1 Training

- Our model was trained on FER2013 dataset (35887 images in total: 28709 for training, 3589 for validation, 3589 for testing).
- We used standard data augmentation to balance the dataset for each emotional expression.
- Adam, the stochastic gradient-based optimizer, learning rate=0.001, categorical cross entropy loss were used
- Training was performed in up to 75 epochs with batch size = 128
- Accuracy (Calculates how often predictions equal labels) is a metric

B.1.2 Model Architecture

The model has a total of thirteen layers: six convolutional layers, three pooling layers, one flatten, and three fully connected layers. The inputs are grayscale images that are cropped to 48x48 shape. The output is 6 confidence scores representing the probability distribution of 6 basic emotions.

B.2 Fine-tuned VGGnet

For cross dataset experiments VGGnet gets fine-tuned with all used datasets (JAFFE, TFEID, TFED, CK+). As a result, there 5 versions of VGGnet:

- VGGnet trained on FER2013 without fine-tuning
- VGGnet trained on FER2013 fine-tuned with JAFFE
- VGGnet trained on FER2013 fine-tuned with TFEID
- VGGnet trained on FER2013 fine-tuned with TFED
- VGGnet trained on FER2013 fine-tuned with CK+

B.2.1 Training

Weights from VGGnet except for the last layer (fully connected dense layer) for classification are transferred. The learning rate was set to 0.0001, training was performed over 100 epochs with batch size 16. The outputs are again 6 confidence scores representing the probability distribution of 6 basic emotions. The data set split was 80% of samples for training and 20% of samples for evaluation.

B.3 Micro-Emotion Detector from Video

B.3.1 Training

The convolutional part of the 3DCNN was pre-trained on SMIC. The SMIC dataset consists of a total of 164 micro-expression videos of three categories: surprise, positive and negative. For learning the classifier we used the SGD optimizer, learning rate=0.01, categorical cross entropy loss. The model was trained for 100 epochs with batch size = 16. The measure of accuracy (Calculates how often predictions equal labels) is a metric.

B.3.2 Model Architecture

The model contains one 3d convolutional layer, one pooling layer, one flattening and two fully connected dense layers. The input during training is 18 frames of 64x64 grayscale image. The output is 3 confidence values representing the probability distribution of 3 emotions: positive, negative, surprised.

B.4 Fine-tuned Micro-Emotion Detector from Video

For cross dataset evaluations the 3DCNN gets fine-tuned with all used datasets (JAFFE, TFEID, TFED, CK+). As a result, there are 5 versions of 3DCNN:

1. 3DCNN trained on SMIC without fine-tuning
2. 3DCNN trained on SMIC fine-tuned with JAFFE
3. 3DCNN trained on SMIC fine-tuned with TFEID
4. 3DCNN trained on SMIC fine-tuned with TFED
5. 3DCNN trained on SMIC fine-tuned with CK+

The model 1 is used as 3DCNN and model 5 as 3DCNN+ in the experimental work. The model 1 is used as input to the MLP when using the 3DCNN and the model 5 is used when the 3DCNN+ is used. The outputs are four points (probability distribution of 4 emotions: positive, negative, surprised, neutral)

B.4.1 Training

Weights from 3DCNN except for the last layer (fully connected dense layer) for classification are transferred. The last dense layer has 4 units as the model has 4 classes. Stochastic gradient descent is used for optimization with learning rate=0.01, trained over 100 epochs and with batch size of 16.

The input to the 3DCNN/3DCNN+ is expected to be a sequence of 18 images. As JAFFE, TFEID, TFED, CK+ datasets contain facial expression images (no video), getting a sequence of 18 frames is impossible. Instead, a copy of the same image is used 18 times to get a depth of 18 for input. The output of the fine tuned models was always 4 points (probability distribution of 4 basic emotions: positive, negative, neutral, surprised). Finally 20% of data was used for testing and 80% for training.

B.5 Action Units Detector

To extract AUs, OpenFace 2.0 [5] was used. Only information about 11 Action Units are recorded: (1, 2, 4, 5, 6, 7, 12, 15, 20, 23, 26) even though 18 Action Units are detected. 11 points represent intensity of a specific action unit Model used in the software: linear kernel Support Vector Regression. Action Units were detected for a single image at a time for all 4 datasets.

B.6 Image Features

The features extractor are obtained from a VGG-11 pre-trained on ImageNet. The input images are transformed to grayscale before processing however the original shape is preserved. The output of the network is 2752 image features.

Appendix C Datasets

C.1 Dataset Description

Facial Expression Recognition 2013 Dataset (FER2013)

FER2013 is one of the most widely used benchmarks. It consists of 35887 images. We have not included the "disgust" class as it is usually accepted as less important. Moreover, this class contains the fewest number of images and makes the dataset less balanced. FER2013 was used to train the VGG16 model to extract CNN-based emotion predictions.

Spontaneous Micro-expression Database (SMIC)

The dataset consists of 164 videos from 16 different subjects. There are 3 classes: positive, negative, and surprise. 18 frames from each video were cut and used in our study as a sequence of images, not single frames. The SMIC dataset was used to pre-train the 3D CNN to detect micro-expressions.

The Extended Cohn-Kanade Dataset (CK+)

The dataset contains 593 video sequences from 123 subjects, where videos start from neutral expression and end with the peak of a specific emotion. For our experiments, however, we extracted a sequence of three images during emotional peak and some frames for the "neutral" class. In total, 838 images in 6 emotions are used for fine-tuning the 3D micro model and test MLP.

Japanese Female Facial Expression Dataset (JAFFE)

The JAFFE is a dataset with 213 images from 10 Japanese females with posed expressions. There are seven emotions, however, we have not used the "disgust" class in our experiment.

Tsinghua facial expression Dataset (TFED)

The dataset consists of images of 110 Chinese people of different ages and gender. There are eight facial emotional expressions, where 6 of them have been used in this study.

Taiwanese facial expression image Dataset (TFEID)

The TFEID database consists of 48 subjects performing eight emotions. For our experiment, 6 emotions have been used, in total 228 images.

C.2 Datasets Summary

Table C2 Table summarizing the information on the used datasets

dataset	demography	# of images	Train set	Test set
tfed	47 older (21 male and 26 female) and 63 young (32 male and 31 female). In total, 53 male and 57 female. All Chinese. 35 young and 35 older face rater participants took part in the rating task.	660	528	132
jaffe	10 Japanese female expressers, 6 basic facial expressions, Each image has averaged semantic ratings on 6 facial expressions by 60 Japanese viewers	184	147	37
tfeid	40 Taiwanese models	228	182	46
ck+	139 adults. Participants were 18 to 50 years of age, 69% female, 81% Euro-American, 13% Afro-American, and 6% other groups	838	670	168
fer2013	No info on ethnicity & gender distribution. FER-2013 database was introduced in the ICML 2013 Challenges in Representation Learning [1]. The database was created using the Google image search API and faces have been automatically registered. Faces are labeled as any of the six basic expressions as well as the neutral.	35887	28709	3589
SMIC (micro-expression)	164 micro-expression clips from 16 participants (mean age is 28.1 years, six females and ten males, eight Caucasians and eight Asians).	164	132	32

References

[1] C. Pramerdorfer and M. Kampel, "Facial expression recognition using convolutional neural networks: State of the art," *CoRR*, vol. abs/1612.02903, 2016. [Online]. Available: http://arxiv.org/abs/1612.02903

[2] Y. Khaireddin and Z. Chen, "Facial emotion recognition: State of the art performance on FER2013," *CoRR*, vol. abs/2105.03588, 2021. [Online]. Available: https://arxiv.org/abs/2105.03588

[3] G. P. Kusuma, J. Jonathan, and A. P. Lim, "Emotion recognition on fer-2013 face images using fine-tuned vgg-16," *Advances in Science, Technology and Engineering Systems Journal*, vol. 5, pp. 315–322, 2020.

[4] T. B. Abdallah, I. Elleuch, and R. Guermazi, "Student behavior recognition in classroom using deep transfer learning with vgg-16," *Procedia Computer Science*, vol. 192, pp. 951–960, 2021, knowledge-Based and Intelligent Information & Engineering Systems: Proceedings of the 25th International Conference KES2021. [Online]. Available: https://www.sciencedirect.com/science/article/pii/S1877050921015866

[5] T. Baltrušaitis, A. Zadeh, Y. C. Lim, and L.-P. Morency, "Openface 2.0: Facial behavior analysis toolkit," in *IEEE International Conference on Automatic Face and Gesture Recognition*, 2018.
